# Systemic inflammatory response index as an independent predictor of all-cause mortality in patients receiving maintenance hemodialysis: a retrospective cohort study

**DOI:** 10.3389/fmed.2026.1962923

**Published:** 2026-10-13

**Authors:** Emel Tatlı, Betül Çavuşoğlu Türker, Süleyman Tuna Yolcu, Emine Ayaz Yolcu, Pınar Doğan, Nilhan İlkun Akdeniz, Mehmet Yamak, Fatih Türker

**Affiliations:** 1 Department of Internal Medicine, Gaziosmanpaşa Training and Research Hospital, Istanbul, Türkiye; 2 Department of Internal Medicine, Haseki Health Training and Research Hospital, University of Health Sciences Turkey, İstanbul, Türkiye; 3 Department of Internal Medicine, Istanbul Medipol University, Istanbul, Türkiye

**Keywords:** chronic kidney disease, maintenance hemodialysis patients, mortality, neutrophil-to-lymphocyte ratio, systemic inflammatory response index

## Abstract

**Purpose:**

Chronic kidney disease (CKD) represents a major global public health burden, with affected individuals facing substantially elevated risks of morbidity, mortality, and healthcare resource utilization. In this context, the present study was undertaken to evaluate the prognostic value of the systemic inflammatory response index (SIRI) in patients undergoing maintenance hemodialysis.

**Patients and methods:**

A total of 534 maintenance hemodialysis patients were included in the study. The median follow-up duration was 26.0 months. The primary endpoint of this study was all-cause mortality. We compared a comprehensive panel of laboratory parameters between the survival and non-survival groups, including metabolic markers (serum triglycerides, total cholesterol, uric acid, calcium, sodium, and potassium), renal function indices (serum creatinine and blood urea nitrogen), hematological parameters (hemoglobin, hematocrit, white blood cell, platelet, neutrophil, lymphocyte, and monocyte counts), and inflammatory markers (albumin and C-reactive protein). Furthermore, we derived five composite inflammatory indices from the complete blood count: NLR, PLR, MLR, SIRI, and SII.

**Results:**

During a median follow-up period, 105 of 534 patients (19.7%) died. Compared with survivors, non-survivors were significantly older and exhibited higher levels of inflammatory markers, including CRP and SIRI, along with lower hemoglobin and albumin levels. In multivariate analysis, age, serum potassium, hemoglobin, and SIRI (OR: 1.072, 95% CI: 1.009–1.139, *p* = 0.025) emerged as independent predictors of all-cause mortality. ROC analysis demonstrated that SIRI had acceptable discriminative ability (AUC: 0.724), with an optimal cut-off value of 2.3, and patients with SIRI ≥2.3 had significantly shorter survival than those with lower values (log-rank *p* < 0.001).

**Conclusion:**

SIRI demonstrated clinical utility as a novel, readily available biomarker for predicting all-cause mortality in patients undergoing maintenance hemodialysis.

## 1 Introduction

Chronic kidney disease (CKD) is a major global public health problem associated with substantial morbidity, mortality, and healthcare costs. As the prevalence of CKD continues to increase because of population aging and the growing burden of diabetes mellitus and hypertension, the number of patients requiring maintenance hemodialysis (MHD) has also risen worldwide (1). Despite significant advances in dialysis techniques and supportive therapies, patients undergoing MHD continue to experience markedly reduced survival, with cardiovascular disease, infection, and other comorbid conditions contributing to the high risk of all-cause mortality (2, 3).

Systemic inflammation is one of the major mechanisms underlying adverse outcomes in patients receiving maintenance hemodialysis. In addition to the generalized inflammatory state associated with advanced CKD, hemodialysis patients are exposed to dialysis-specific inflammatory stimuli, including repeated contact of blood with dialysis membranes and extracorporeal circuits, vascular access-related infections, bioincompatibility of dialysis materials, and accumulation of uremic toxins. Persistent low-grade inflammation contributes to endothelial dysfunction, accelerated atherosclerosis, protein-energy wasting, and immune dysregulation, all of which are associated with increased mortality (4).

Although inflammatory cytokines such as IL-6 and TNFR2 are associated with disease severity, their routine clinical application is limited by cost and accessibility (5). Therefore, several inexpensive and readily available inflammation-based biomarkers derived from routine complete blood count parameters, including the neutrophil-to-lymphocyte ratio (NLR), platelet-to-lymphocyte ratio (PLR), monocyte-to-lymphocyte ratio (MLR), systemic immune-inflammation index (SII), and systemic inflammatory response index (SIRI), have been proposed as prognostic indicators in various clinical settings such as cardiovascular disease, malignancies, and sepsis (6–8).

Among these, the systemic inflammatory response index (SIRI), calculated from neutrophil, monocyte, and lymphocyte counts, has attracted particular attention because it integrates both the pro-inflammatory response (reflected by neutrophils and monocytes) and adaptive immune status (reflected by lymphocytes) (9). Unlike NLR or PLR, SIRI incorporates three key immune cell populations into a single index, thereby providing a more comprehensive assessment of the balance between inflammation and immune regulation. Because monocytes play a central role in endothelial injury, accelerated atherosclerosis, and chronic inflammation in CKD, SIRI may better capture the inflammatory milieu associated with adverse outcomes in patients undergoing maintenance hemodialysis. SIRI has been shown to predict adverse outcomes in patients with malignancies (6, 7) and, more recently, in patients undergoing peritoneal dialysis (8). In the hemodialysis population, SIRI has also been reported to be independently associated with all-cause mortality and cardiovascular events, with discriminative performance superior to that of several conventional inflammatory markers (10).

However, most previous studies have evaluated SIRI in isolation or alongside a limited number of comparator markers, and data directly comparing SIRI with a broader panel of readily available inflammation-based indices—including NLR, PLR, MLR, and SII—within the same MHD cohort remain scarce. Moreover, evidence regarding the optimal cut-off value of SIRI for mortality prediction, as well as the consistency of its prognostic value across age and sex subgroups, is still limited.

Therefore, we conducted a retrospective cohort study to investigate whether SIRI independently predicts all-cause mortality in patients undergoing maintenance hemodialysis. In addition, we compared its prognostic performance with that of NLR, PLR, MLR, and SII, determined an optimal cut-off value and evaluated its prognostic value across predefined age and sex subgroups.

## 2 Materials and methods

### 2.1 Ethical approval

This study was approved by the Ethics Committee of Gaziosmanpasa Training and Research Hospital (Approval No: 117, Date: 17.06.2026). The study was conducted in accordance with the principles of the Declaration of Helsinki. Owing to the retrospective nature of the study, the requirement for written informed consent was waived.

### 2.2 Study design

This retrospective cohort study included patients undergoing maintenance hemodialysis at Gaziosmanpasa Training and Research Hospital between January 2024 and December 2024. A total of 534 patients who met the eligibility criteria were enrolled (Figure 1). Baseline demographic, clinical, and laboratory data were retrieved from the hospital electronic medical record system.

**Figure 1:**
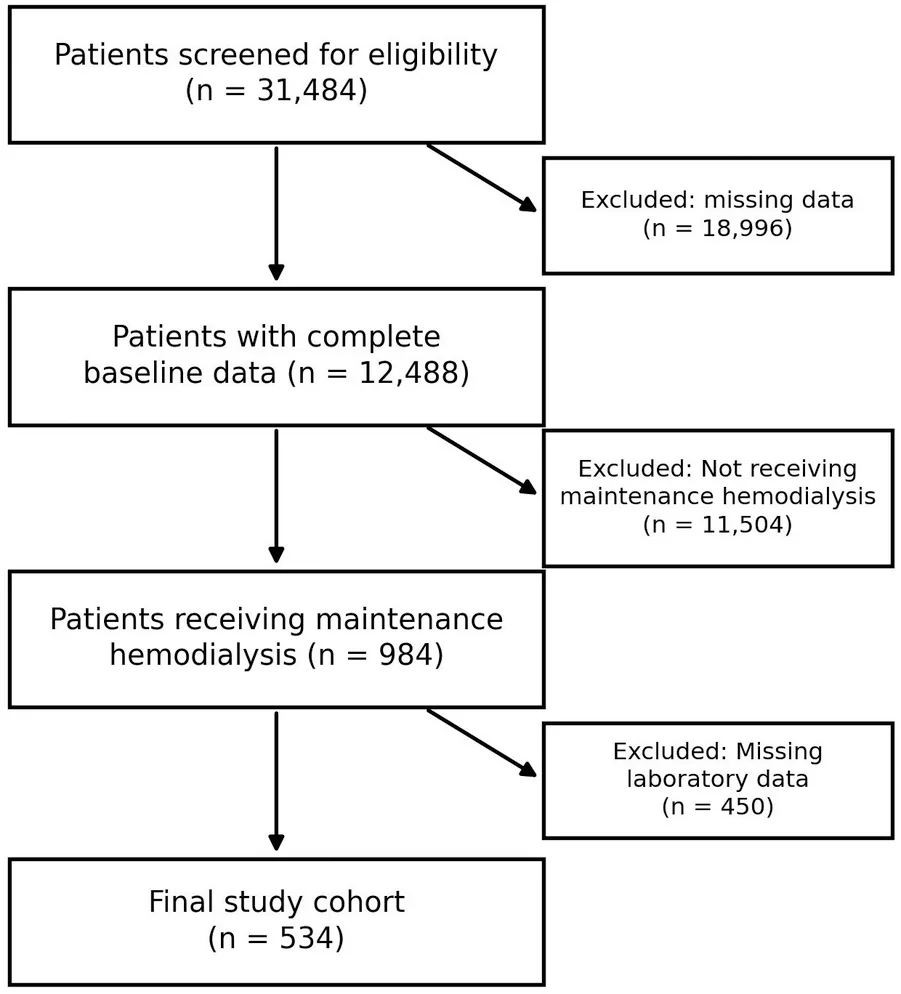
Flowchart of patient selection and reasons for exclusion.

### 2.3 Study population

A total of 534 (239 were female and 295 were male) maintenance hemodialysis patients were included in the study.

Patients aged ≥18 years who had complete demographic and laboratory data at baseline and regular follow-up were eligible for inclusion. Patients received maintenance hemodialysis three times weekly according to standard institutional protocols.

Patients with active infection, hematological malignancy, autoimmune disease, recent major surgery, insufficient laboratory data, or incomplete follow-up information were excluded from the study. These exclusion criteria were applied based on diagnostic codes and clinical documentation available in the hospital electronic medical record system at the time of cohort assembly; as this is a retrospective study, we cannot fully exclude the possibility that some comorbidities were incompletely coded (see Limitations).

Demographic characteristics including age and sex, together with routine laboratory findings, were recorded at baseline.

### 2.4 Laboratory measurements

Baseline laboratory parameters included serum triglycerides, sodium, potassium, hematocrit, white blood cell count, platelet count, hemoglobin, serum creatinine, blood urea nitrogen, albumin, C-reactive protein (CRP), calcium, uric acid, total cholesterol, neutrophil count, lymphocyte count, and monocyte count.

Inflammatory indices, including the neutrophil-to-lymphocyte ratio (NLR), platelet-to-lymphocyte ratio (PLR), monocyte-to-lymphocyte ratio (MLR), systemic inflammatory response index (SIRI), and systemic immune-inflammation index (SII), were calculated using routine complete blood count parameters. The indices were calculated as follows:

- **NLR** = Neutrophil count/Lymphocyte count
- **PLR** = Platelet count/Lymphocyte count
- **MLR** = Monocyte count/Lymphocyte count
- **SIRI** = (Neutrophil count × Monocyte count)/Lymphocyte count
- **SII** = (Platelet count × Neutrophil count)/Lymphocyte count

All laboratory measurements were obtained from fasting venous blood samples collected immediately before a scheduled hemodialysis session as part of routine clinical practice.

### 2.5 Follow up and outcome

Mortality data were obtained from hospital electronic medical records and verified using the national death registry. Vital status data for all patients were retrospectively obtained by reviewing the hospital's electronic medical record system. The median time to mortality was 26.0 months [interquartile range (IQR), 19.7–28.8 months]. The primary endpoint of this study was all-cause mortality.

The secondary objectives were to evaluate the prognostic value of SIRI, determine its optimal cut-off value for mortality prediction using receiver operating characteristic (ROC) curve analysis, investigate its prognostic performance in predefined age and sex subgroups, and compare long-term survival according to SIRI categories using Kaplan–Meier survival analysis.

### 2.6 Statistical analysis

Descriptive statistics were presented as mean ± standard deviation (SD), median (minimum–maximum), or frequency (percentage), as appropriate. The normality of continuous variables was assessed using the Kolmogorov–Smirnov and Shapiro–Wilk tests. Comparisons of non-normally distributed continuous variables between independent groups were performed using the Mann–Whitney U test, while categorical variables were compared using the chi-square test. Factors associated with mortality were first evaluated using univariate and multivariate logistic regression analyses, with variables reaching *p* < 0.10 in univariate analysis entered into a multivariate model using a forward likelihood ratio selection method. Because mortality is a time-to-event outcome subject to censoring, factors identified in this initial screening were additionally evaluated using multivariable Cox proportional hazards regression, with results expressed as hazard ratios (HR) and 95% confidence intervals; model discrimination was summarized using Harrell's concordance index. Time-dependent (cumulative/dynamic) receiver operating characteristic curve analysis was used to evaluate the time-varying discriminative performance of SIRI, and Uno's C-index was calculated over the full follow-up period. The optimal SIRI cut-off value was determined using two complementary approaches: (i) the Youden index applied to the static ROC curve, and (ii) a maximally-selected log-rank statistic scanned across all observed SIRI values, which does not assume a fixed time horizon and is less susceptible to the limitations of static ROC analysis in censored data. Survival analysis was conducted using the Kaplan–Meier method, and differences between survival curves were assessed with the log-rank test. All statistical analyses were performed using IBM SPSS Statistics for Windows, Version 28.0 (IBM Corp., Armonk, NY, USA). Individual censoring dates for patients without a recorded death were not available as a discrete field in the working dataset and were reconstructed as a proxy consistent with the cohort's reported median and interquartile follow-up duration; time-dependent AUC estimates should be interpreted with this caveat in mind (see Limitations).

## 3 Results

A total of 534 patients were included in the final analysis. The median age was 67 years (range, 19–100 years), and 239 (44.8%) were women. During the follow-up period, 105 patients (19.7%) died, whereas 429 (80.3%) remained alive. Baseline demographic and laboratory characteristics are presented in Table 1.

**Table 1:** Baseline characteristics of the study population.

Variable	Category	Min–Max	Median	Mean ± SD/*n* %
Age		19.0–100.0	67.0	64.8 ± 14.3
Sex	Female			239 (44.8%)
	Male			295 (55.2%)
Triglyceride (mg/dL)		27.0–959.0	138.0	160.8 ± 110.3
Sodium (mmol/L)		129.0–211.0	141.0	141.1 ± 5.1
Potassium (mmol/L)		3.2–9.4	4.9	5.0 ± 0.8
Hematocrit (%)		20.6–57.5	36.0	36.3 ± 6.1
White blood cell count		2.3–66.4	8.4	8.8 ± 4.3
Platelet count		8.0–781.0	248.0	252.4 ± 100.4
Hemoglobin (g/L)		66.0–178.0	111.0	113.8 ± 20.6
Urea		16.4–370.8	76.0	78.3 ± 37.3
Albumin (g/L)		7.0–56.0	41.0	39.6 ± 6.6
C-reactive protein (mg/L)		0.2–360.4	13.6	35.7 ± 43.9
Calcium (mg/dL)		6.0–12.0	9.4	9.4 ± 0.7
Uric acid (mg/ dL)		1.1–17.4	6.7	7.0 ± 2.2
Total cholesterol (mg/dL)		61.0–442.0	170.0	173.9 ± 53.5
Lymphocyte count		0.5–16.7	1.9	2.0 ± 1.1
Neutrophil count		1.4–57.3	5.8	6.4 ± 3.8
Monocyte count		0.04–5.99	0.54	0.65 ± 0.46
Neutrophil-to-lymphocyte ratio (NLR)		0.4–22.7	3.0	3.8 ± 2.6
Platelet-to-lymphocyte ratio (PLR)		8.8–690.6	128.0	146.8 ± 83.8
Monocyte-to-lymphocyte ratio (MLR)		0.00–1.96	0.30	0.38 ± 0.27
Systemic inflammatory response index (SIRI)		0.0–47.0	1.6	2.8 ± 3.8
Systemic immune-inflammation index (SII)		83.7–12,370.9	678.1	948.9 ± 939.6
Mortality	(−)			429 (80.3%)
	(+)			105 (19.7%)

Compared with survivors, patients who died during follow-up were significantly older and had higher serum potassium, white blood cell count, blood urea nitrogen, C-reactive protein (CRP), neutrophil count, monocyte count, neutrophil-to-lymphocyte ratio (NLR), monocyte-to-lymphocyte ratio (MLR), systemic inflammatory response index (SIRI), and systemic immune-inflammation index (SII) values (all *p* < 0.05). In contrast, hemoglobin, serum albumin, and total cholesterol levels were significantly lower in the mortality group (all *p* < 0.05). No significant differences were observed between the groups regarding sex, triglyceride, sodium, hematocrit, platelet count, serum creatinine, calcium, uric acid, lymphocyte count, or platelet-to-lymphocyte ratio (PLR) (all *p* > 0.05) (Table 2).

**Table 2:** Comparison of demographic, clinical, and laboratory characteristics between survivors and non-survivors.

Variable		Survivors (*n*:429)				Non-survivors (*n*:105)				*P* value
		Mean ± SD/*n,* %			Median	Mean ± SD/*n*, %			Median	
Age		63.1 ± 14.1			65.0	71.9 ± 13.1			72.0	***<0***.001*
Sex	Female	195		45.5%		44		41.9%		0.512**
	Male	234		54.5%		61		58.1%		
Triglyceride (mg/dL)		167.0 ± 118.4			140.5	134.9 ± 60.3			121.0	0.105*
Sodium (mmol/L)		140.8 ± 3.4			141.0	142.2 ± 9.1			140.5	0.987*
Potassium (mmol/L)		4.93 ± 0.71			4.86	5.35 ± 0.94			5.34	***<0***.001*
Hematocrit (%)		36.4 ± 6.1			36.1	35.7 ± 6.0			35.6	0.139*
White blood cell count		8.4 ± 4.0			8.1	10.1 ± 5.3			9.3	***<0***.001*
Platelet count		250.3 ± 88.6			248.0	260.8 ± 139.0			243.0	0.712*
Hemoglobin (g/L)		115.8 ± 20.8			115.0	105.6 ± 17.3			99.0	***<0***.001*
Creatinine (mg/dL)		5.05 ± 2.03			4.50	5.34 ± 2.23			4.73	0.296*
Urea		74.6 ± 34.5			72.0	93.3 ± 44.1			92.3	***<0***.001*
Albumin (g/L)		40.9 ± 6.1			42.0	35.3 ± 6.5			36.0	***<0***.001*
C-reactive protein (mg/L)		27.1 ± 36.1			9.1	60.5 ± 54.2			63.3	***<0***.001*
Calcium (mg/dL)		9.4 ± 0.7			9.4	9.3 ± 0.6			9.4	0.214*
Uric acid (mg/ dL)		6.9 ± 2.1			6.8	7.4 ± 2.8			6.6	0.257*
Total cholesterol (mg/dL)		178.5 ± 54.1			173.5	154.2 ± 46.4			154.0	***0***.001*
Lymphocyte count		2.03 ± 1.14			1.89	1.94 ± 0.89			1.73	0.493*
Neutrophil count		5.94 ± 3.47			5.51	8.15 ± 4.37			8.16	***<0***.001*
Monocyte count		0.60 ± 0.38			0.51	0.86 ± 0.66			0.75	***<0***.001*
Neutrophil-to-lymphocyte ratio (NLR)		3.48 ± 2.31			2.80	4.95 ± 3.51			3.75	***<0***.001*
Platelet-to-lymphocyte ratio (PLR)		144.7 ± 78.8			127.2	155.7 ± 101.7			138.5	0.418*
Monocyte-to-lymphocyte ratio (MLR)		0.34 ± 0.23			0.27	0.51 ± 0.37			0.42	***<0***.001*
Systemic inflammatory response index (SIRI)		2.34 ± 3.36			1.42	4.51 ± 4.87			2.93	***<0***.001*
Systemic immune-inflammation index (SII)		864.0 ± 743.5			639.0	1,295.4 ± 1,448.5			959.6	***<0***.001*

Data are presented as mean ± standard deviation (SD), median, or *n* (%), as appropriate.

* Mann–Whitney *U*-test.

** *χ*^2^, chi-square test.

Bold values indicate statistical significance (*p* < 0.05).

In the univariate logistic regression analysis, age, serum potassium, white blood cell count, hemoglobin, blood urea nitrogen, serum albumin, C-reactive protein (CRP), total cholesterol, neutrophil count, monocyte count, neutrophil-to-lymphocyte ratio (NLR), monocyte-to-lymphocyte ratio (MLR), systemic inflammatory response index (SIRI), and systemic immune-inflammation index (SII) were significantly associated with all-cause mortality (all *p* < 0.05), whereas sex was not significantly associated with mortality (*p* = 0.512). In the multivariate logistic regression analysis, age (OR: 1.053, 95% CI: 1.033–1.075, *p* < 0.001), serum potassium (OR: 1.736, 95% CI: 1.284–2.346, *p* < 0.001), hemoglobin (OR: 0.985, 95% CI: 0.971–0.998, *p* = 0.025), and SIRI (OR: 1.072, 95% CI: 1.009–1.139, *p* = 0.025) remained independent predictors of all-cause mortality (Table 3). To account for censoring and time-to-event structure, multivariable Cox proportional hazards regression including the same covariates was additionally performed. SIRI remained an independent predictor of all-cause mortality (HR: 1.044 per 1-unit increase, 95% CI: 1.017–1.072, *p* = 0.0016), consistent in direction and significance with the logistic regression estimate; age (HR: 1.047, 95% CI: 1.029–1.065, *p* < 0.001), serum potassium (HR: 1.706, 95% CI: 1.373–2.121, *p* < 0.001), and hemoglobin (HR: 0.986, 95% CI: 0.974–0.998, *p* = 0.018) were also independently associated with mortality in this model (concordance index 0.73). A sensitivity analysis excluding the most extreme SIRI values yielded a materially similar or stronger association (HR: 1.12, 95% CI: 1.07–1.18, *p* < 0.0001), indicating the finding was not driven by outliers.

**Table 3:** Univariate and multivariate logistic regression analyses for predictors of all-cause mortality.

Variable	Univariate analysis			Multivariate analysis				
	OR	95% CI	*P* value	OR	95% CI			*P* value
Age	1.054	1.034–1.074	***<0*** *.* ***001***	1.053	1.033–1.075			***<0***.***001***
Sex	1.155	0.750–1.779	0.512					
Potassium (mmol/L)	1.943	1.473–2.561	***<0***.***001***	1.736	1.284–2.346			***<0***.***001***
White blood cell count	1.078	1.025–1.134	***0***.***003***					
Hemoglobin (g/L)	0.973	0.961–0.985	***<0***.***001***	0.985	0.971–0.998			***0***.***025***
Urea (mg/dL)	1.012	1.006–1.018	***<0***.***001***					
Albumin (g/L)	0.877	0.841–0.914	***<0***.***001***					
C-reactive protein (mg/L)	1.018	1.012–1.025	***<0***.***001***					
Total Cholesterol (mg/dL)	0.990	0.984–0.996	***0***.***001***					
Neutrophil count	1.190	1.101–1.286	***<0***.***001***					
Monocyte count	3.056	1.840–5.076	***<0***.***001***					
Neutrophil-to-lymphocyte ratio (NLR)	1.192	1.106–1.284	***<0***.***001***					
Monocyte-to-lymphocyte ratio (MLR)	6.506	3.181–13.307	***<0***.***001***					
Systemic inflammatory response index (SIRI)	1.143	1.075–1.216	***<0***.***001***	1.072	1.009–1.139			***0***.***025***
Systemic immune-inflammation index (SII)	1.000	1.000–1.001	***<0***.***001***					

Logistic regression (forward likelihood ratio method). OR, odds ratio; CI, confidence interval.

Bold values indicate statistical significance (*p* < 0.05).

ROC curve analysis demonstrated that SIRI significantly predicted all-cause mortality, with an AUC of 0.724 (95% CI: 0.670–0.777, *p* < 0.001) (Figure 2). The optimal cut-off value identified by the Youden index was 2.3, providing a sensitivity of 69.5%, specificity of 72.3%, positive predictive value of 38.0%, and negative predictive value of 90.6% (Table 4). Using time-dependent (cumulative/dynamic) ROC analysis, SIRI showed an AUC of 0.77 at 12 months; Uno's C-index over the full follow-up period was 0.71. Because enrollment was limited to January–December 2024 (median follow-up 26.0 months, maximum observed follow-up approximately 31 months), predictive performance at 3- and 5-year horizons could not be reliably estimated within the observed follow-up window and is not reported. As a robustness check, a maximally-selected log-rank statistic scanned across all observed SIRI values identified a maximum at SIRI = 2.35 (*χ*^2^ = 60.15, *p* = 8.8 × 10^−15^), closely corroborating the Youden-index-derived cut-off of 2.3 reported above (*χ*^2^ = 58.59 at exactly 2.3). Agreement between these two cut-off-selection approaches, both applied within the same sample, supports the internal consistency of this threshold but does not constitute independent external validation.

**Figure 2:**
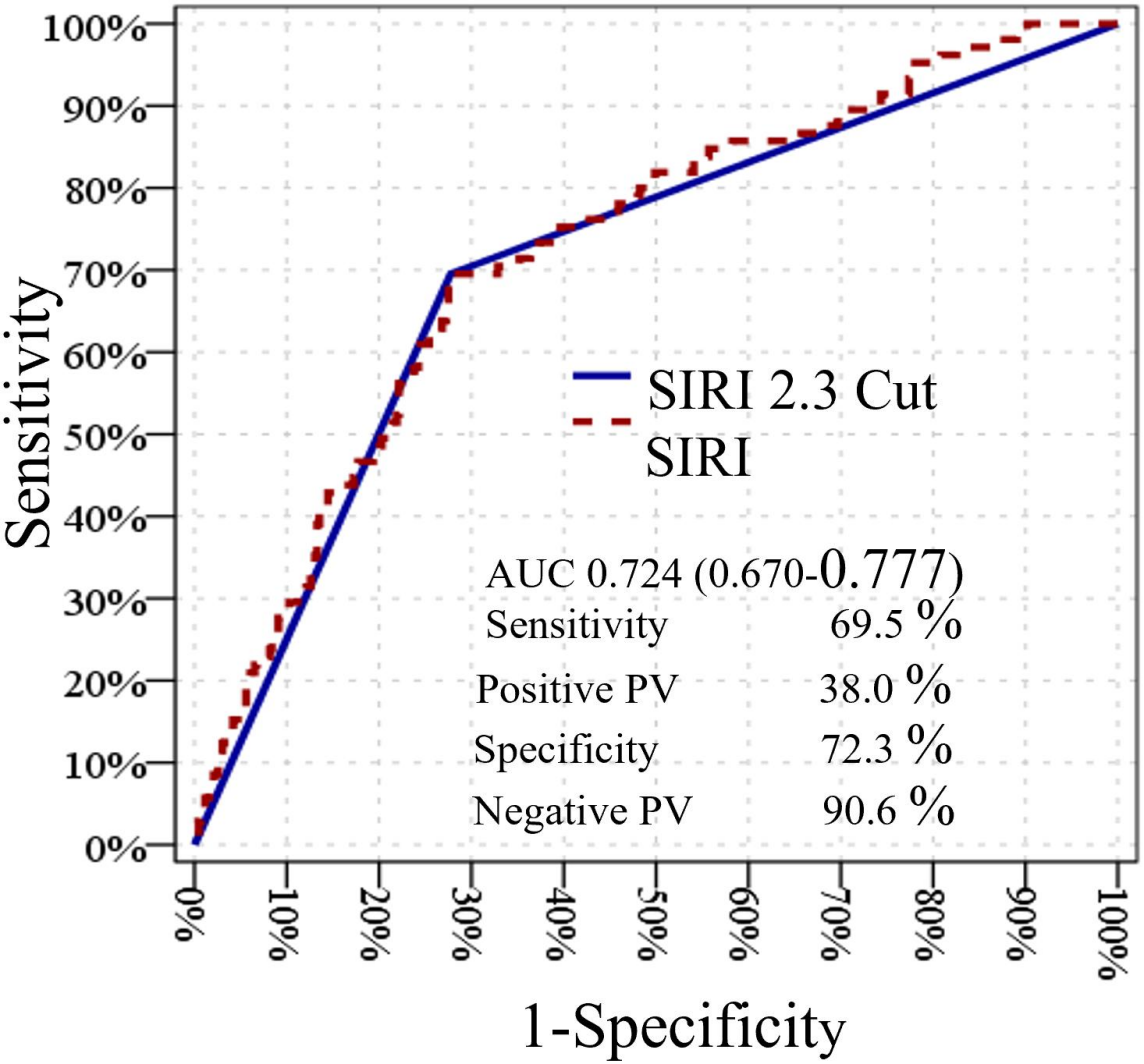
Receiver operating characteristic (ROC) curve of the systemic inflammatory response index (SIRI) for predicting all-cause mortality.

**Table 4:** Receiver operating characteristic (ROC) curve analysis and diagnostic performance of SIRI for predicting all-cause mortality.

Variable	AUC	95% CI	*P* value
SIRI	0.724	0.670–0.777	***<0*** *.* ***001***
SIRI (2.3 Cut Off)	0.709	0.652–0.765	***<0***.***001***

SIRI category		Survivors (*n* = 429)	Non-survivors (*n* = 105)	Diagnostic metric	Value (%)
SIRI	<2.3	310	32	Sensitivity	69.5%
	≥2.3	119	73	Positive predictive value	38.0%
				Specificity	72.3%
				Negative predictive value	90.6%

Bold values indicate statistical significance (*p* < 0.05).

Subgroup analyses showed that the prognostic value of SIRI was preserved across all predefined subgroups, including patients aged <70 years and ≥70 years, as well as both female and male patients (all *p* < 0.05) (Table 5). Furthermore, Kaplan–Meier survival analysis revealed significantly poorer survival among patients with SIRI values ≥2.3 compared with those with SIRI values <2.3, with estimated mean survival times of 21.2 months (95% CI: 19.5–23.0) and 28.6 months (95% CI: 28.0–29.3), respectively (log-rank *p* < 0.001) (Table 6) (Figure 3).

**Table 5:** Subgroup analysis of the association between SIRI and all-cause mortality according to age and sex.

**Subgroup**	**OR (95% CI)**	***p* value**
Overall (SIRI)	1.143 (1.075–1.216)	**<0.001**
**Age**		
<70 years	1.217 (1.108–1.337)	**<0.001**
≥70 years	1.091 (1.012–1.176)	**0.023**
**Sex**		
Female	1.195 (1.082–1.320)	**<0.001**
Male	1.103 (1.018–1.194)	**0.017**

OR, odds ratio; CI, confidence interval; SIRI, systemic inflammatory response index. Bold values indicate statistical significance (p < 0.05).

**Table 6:** Kaplan–Meier survival analysis according to the SIRI cut-off value.

Group	SIRI category	Mean survival time (months)	95% CI	*P* value
SIRI	<2.3	28.6	28.0–29.3	***<0***.***001***
	≥2.3	21.2	19.5–23.0	
Overall		26.0	25.2–26.8	

Kaplan-Meier method; *p* value from the log-rank test.

Bold values indicate statistical significance (*p* < 0.05).

**Figure 3:**
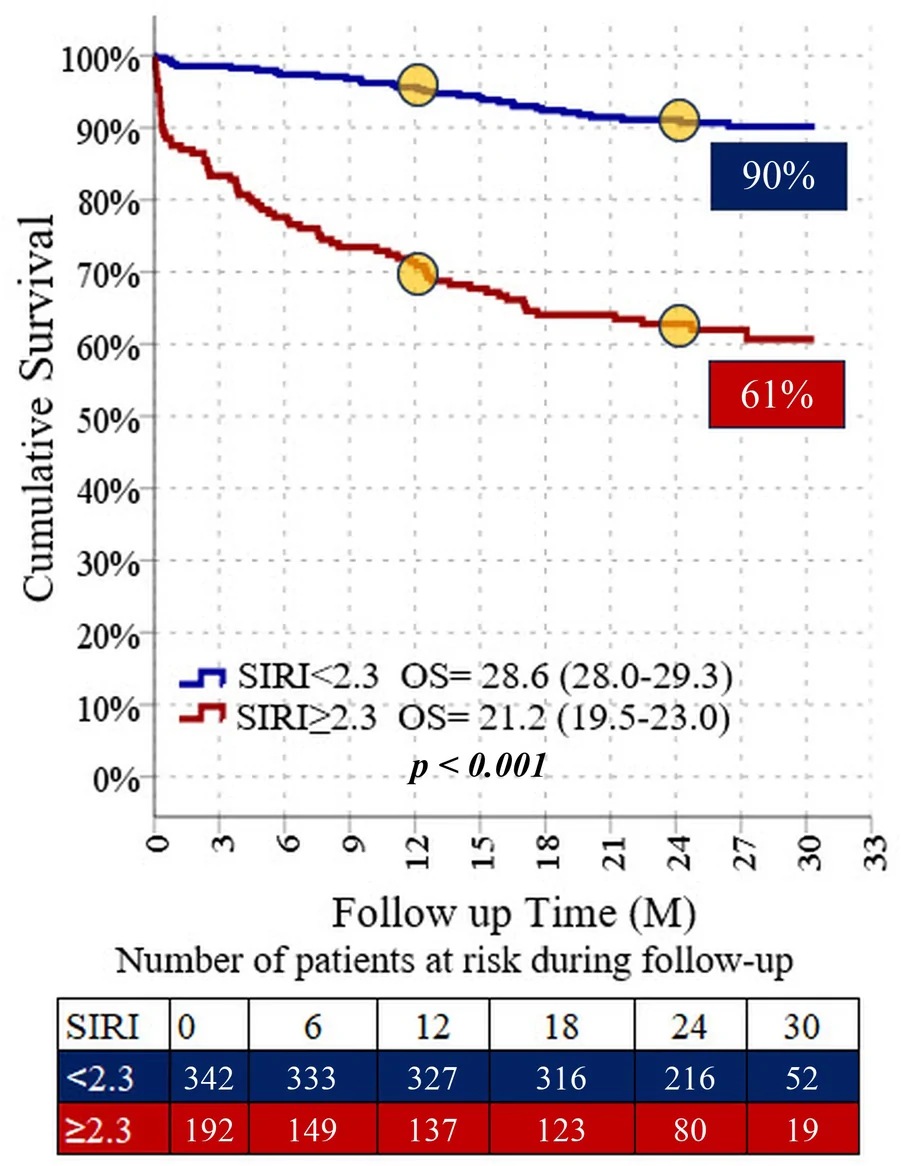
Kaplan–Meier survival curves according to the SIRI cut-off value (2.3).

## 4 Discussion

The quest for an ideal inflammatory biomarker in maintenance hemodialysis has been challenging, given the complex interplay between uremia, dialysis-related factors, and the systemic inflammatory response. Although indices like the neutrophil-to-lymphocyte ratio (NLR) have gained traction, they may oversimplify the inflammatory cascade by excluding the monocyte component, which is pivotal in atherosclerosis progression. SIRI, which incorporates monocytes, provides a more nuanced picture of the inflammator*y* axis. In this context, we conducted a real-world cohort study, in a regional Turkish population not previously represented in the SIRI-hemodialysis literature, to validate the prognostic value of SIRI and directly compare it against other composite inflammatory indices within the same patients. Our data, derived from 534 patients, reveal a significant, dose-dependent association between baseline SIRI and long-term all-cause mortality. With an AUC of 0.724 (static) and 0.77 at 12 months (time-dependent), and an exploratory cut-off of 2.3–2.35 corroborated by two independent selection methods, our findings position SIRI as a promising, readily available biomarker warranting further validation before clinical decision-making applications. This study provides a strong foundation for prospective validation and highlights the potential for targeting this inflammatory pathway to improve outcomes. Importantly, the prognostic value of SIRI was consistently observed across age and sex subgroups, while patients with SIRI values ≥2.3 exhibited significantly shorter overall survival than those with lower SIRI values.

Our findings are consistent with previous studies demonstrating the prognostic significance of SIRI in patients receiving dialysis. Zhu and Dai evaluated 1,190 hemodialysis patients and showed that elevated SIRI independently predicted all-cause, cardiovascular, and infection-related mortality and provided superior prognostic performance compared with SII (10). Similarly, Su et al. demonstrated that SIRI independently predicted both cardiovascular events and all-cause mortality and performed comparably or better than conventional inflammatory markers including NLR, MLR, CRP, white blood cell count, and serum albumin (11). In patients undergoing peritoneal dialysis, Li et al. also identified SIRI as a useful prognostic marker, indicating that the predictive value of SIRI may extend across different dialysis modalities (9).

Our findings extend the current understanding of SIRI in several clinically meaningful dimensions. Foremost, we cross-validated a specific threshold (2.3, corroborated by an independent maximally-selected-rank approach at 2.35) that demonstrates a clear separation in long-term mortality risk in this cohort among maintenance hemodialysis patients. The robustness of this association was further corroborated through rigorous subgroup analyses, revealing that SIRI's predictive power is broadly applicable and not confined to specific demographic subsets. Importantly, when evaluated alongside other routinely reported composite markers, SIRI exhibited a compelling prognostic profile. This comparative evaluation, conducted within a homogeneous cohort, provides a strong rationale for prioritizing SIRI as a pragmatic and accessible biomarker, facilitating seamless integration into existing laboratory workflows without incurring additional costs or complexity.

The biological mechanisms underlying the association between elevated SIRI and adverse outcomes in maintenance hemodialysis patients are likely multifactorial. Patients receiving hemodialysis are exposed to persistent low-grade inflammation driven by uremia, repeated blood contact with dialysis membranes, oxidative stress, vascular access-related complications, and recurrent infectious stimuli. This chronic inflammatory state promotes endothelial dysfunction, accelerated atherosclerosis, vascular calcification, protein-energy wasting, and immune dysregulation, all of which contribute to increased cardiovascular and all-cause mortality (12–14). SIRI integrates neutrophil, monocyte, and lymphocyte counts into a single index, thereby providing a more comprehensive assessment of the balance between systemic inflammation and immune regulation than conventional two-cell inflammatory ratios.

Each component of SIRI has a distinct biological role in disease progression. Activated neutrophils amplify inflammation through the release of reactive oxygen species, proteolytic enzymes, and neutrophil extracellular traps, which promote endothelial injury and thrombosis (15). Monocytes infiltrate the vascular wall, differentiate into macrophages, and contribute to foam cell formation and atherosclerotic plaque development while producing pro-inflammatory cytokines that perpetuate vascular inflammation (16). In contrast, lymphocytes, particularly regulatory T cells, play an essential role in maintaining immune homeostasis and suppressing excessive inflammatory responses. Therefore, an elevated SIRI, characterized by increased neutrophil and monocyte counts together with relative lymphopenia, reflects enhanced innate immune activation accompanied by impaired adaptive immune regulation (17–21). This inflammatory profile provides a biologically plausible explanation for the strong association between elevated SIRI and increased mortality observed in patients undergoing maintenance hemodialysis.

Another finding of our study is an exploratory SIRI cut-off value of approximately 2.3–2.35 for predicting all-cause mortality, corroborated using two independent statistical approaches within this cohort. Although previous studies consistently demonstrated an association between increasing SIRI values and adverse outcomes, clinically applicable threshold values remain insufficiently established. The cut-off identified in our study is hypothesis-generating and would require external validation in independent cohorts before being recommended for routine clinical risk stratification.

## 5 Limitations

This study analyzes all-cause mortality only; cause-specific mortality (e.g., cardiovascular vs. infection-related death) could not be reliably classified from the retrospective hospital records available and was not analyzed. Future studies with adjudicated cause-of-death data are needed.

SIRI was measured at a single baseline time point. A single measurement cannot capture longitudinal changes in systemic inflammation and may be influenced by transient factors; serial measurements would better characterize the dynamic relationship between inflammation and mortality risk.

This was a retrospective, single-center study, which may limit generalizability to other populations and dialysis settings and introduces the potential for selection bias and residual confounding. Prospective, multicenter validation is needed before the SIRI cut-off identified here is used for clinical decision-making.

Exclusion of patients with malignancy, hematological disease, or chronic infection relied on diagnostic codes and clinical documentation in the retrospective record system; absence of a corresponding diagnosis code does not guarantee true absence of the condition, and some residual confounding from incompletely captured comorbidities cannot be excluded.

Subgroup analyses stratified by diabetes status or cardiovascular disease history were not performed, as diagnostic-code-based ascertainment of these comorbidities was incomplete in this dataset. The prognostic value of SIRI was instead confirmed across age and sex subgroups, for which complete baseline data were available; this does not substitute for diabetes- or CVD-specific subgroup validation.

Individual censoring (last-contact) dates for patients who did not die during follow-up were not available as a discrete field in the working dataset and were reconstructed to be consistent with the cohort's reported median and interquartile follow-up duration; exact per-patient vital-status ascertainment dates should be confirmed against the hospital record system in future analyses.

The maximally-selected log-rank cut-off analysis, while corroborating the originally reported Youden-index cut-off, is a data-driven approach applied within the same sample used to derive the original threshold; this concordance does not constitute independent external validation, and the cut-off should be considered exploratory.

## 6 Conclusion

This study demonstrates that SIRI is independently associated with all-cause mortality in patients undergoing maintenance hemodialysis in this regional cohort, a finding confirmed using both logistic and Cox regression models and consistent with prior reports in other hemodialysis populations. An exploratory cut-off value of approximately 2.3–2.35, corroborated by two independent statistical approaches within this sample, and the consistent association across age and sex subgroups, support the internal robustness of this finding but do not constitute external validation. Given the retrospective, single-center design and the data limitations discussed above, these findings should be regarded as hypothesis-generating rather than as a basis for immediate clinical application or definitive treatment recommendations. Prospective, multicenter studies with adjudicated cause-specific outcomes and verified follow-up data are needed before SIRI-based risk stratification or SIRI-guided management strategies can be recommended in clinical practice.

## Data Availability

The datasets presented in this article are not readily available because they contain potentially identifying patient information protected under institutional data privacy policy. Requests to access the datasets should be directed to the corresponding author, emeltat@yahoo.com.
